# Back Propagation Neural Network-Based Predictive Model for Magnetorheological–Chemical Polishing of Silicon Carbide

**DOI:** 10.3390/mi16030271

**Published:** 2025-02-27

**Authors:** Huazhuo Liang, Wenjie Chen, Youzhi Fu, Wenjie Zhou, Ling Mo, Yue Jian, Qi Wen, Dawei Liu, Junfeng He

**Affiliations:** School of Mechatronic Engineering, Guangdong Polytechnic Normal University, Guangzhou 510665, China; lianghuazhuo@gpnu.edu.cn (H.L.); chenwenjie@gpnu.edu.cn (W.C.); yzfu@gpnu.edu.cn (Y.F.); zhouwj@gpnu.edu.cn (W.Z.); ling-mo@163.com (L.M.); jianyue@gpnu.edu.cn (Y.J.); scutwenqi@126.com (Q.W.); liudawei@gpnu.edu.cn (D.L.)

**Keywords:** magnetorheological–chemical polishing, SiC, BP neural network, modelling and prediction

## Abstract

Magnetorheological–chemical-polishing tests are carried out on single-crystal silicon carbide (SiC) to study the influence of the process parameters on the polishing effect, predict the polishing results via a back propagation (BP) neural network, and construct a model of the processing parameters to predict the material removal rate (MRR) and surface quality. Magnetorheological–chemical polishing employs mechanical removal coupled with chemical action, and the synergistic effect of both actions can achieve an improved polishing effect. The results show that with increasing abrasive particle size, hydrogen peroxide concentration, workpiece rotational speed, and polishing disc rotational speed, the MRR first increases and then decreases. With an increasing abrasive concentration and carbonyl iron powder concentration, the MRR continues to increase. With an increasing machining gap, the MRR shows a continuous decrease, and the corresponding changes in surface roughness tend to decrease first and then increase. The prediction models of the MRR and surface quality are constructed via a BP neural network, and their average absolute percentage errors are less than 2%, which is important for the online monitoring of processing and process optimisation.

## 1. Introduction

As a third-generation semiconductor material, single-crystal silicon carbide (SiC) is characterised by a large bandwidth, high breakdown field and thermal conductivity, high radiation resistance, and good chemical stability and is suitable for the production of high-temperature, high-frequency, radiation-resistant, high-power, and high-density integrated electronic devices [1]. The epitaxial process is critical for preparing IC semiconductor devices, which generally requires the surface of a semiconductor substrate to be ultrasmooth, free of defects, free of subsurface damage and with a complete surface lattice to satisfy the requirements for epitaxial film growth [2,3]. However, owing to the characteristics of SiC materials, such as hardness and chemical stability, ultraprecision processing of single-crystalline SiC is difficult [4,5].

The main method for ultrasmooth surface processing of SiC is chemical–mechanical polishing (CMP) [6]. CMP is a complex process involving synergistic chemical and mechanical effects that can be used to achieve ultraprecision polishing of substrates. Via the magnetorheological–chemical-polishing method for SiC, whose removal process is similar to that of CMP, a better result can be obtained [7,8].

Currently, approaches for predictive modelling of the material removal process include physical and data-driven methods. The method for constructing a physical model is based on the physical properties of a target, reflecting the operating mechanism of the target system. For instance, Wu et al. [9] built a material removal rate (MRR) model by considering the polishing pressure and abrasive action and investigated the effects of the abrasive and polishing parameters on the MRR, with a model error within 20%. Zhou et al. [10] investigated the effect of pressure on the polishing process of 4H-SiC in an aqueous environment via molecular dynamics simulations. Liu et al. [11] established a material removal model by using molecular dynamics to study the effects of polishing speed on the atomic motion and structural transformation of a workpiece. Xu et al. [12] established a model for the MRR on the basis of contact mechanics, hydrodynamics, reaction dynamics, wear theory, etc. The simulation results match well with the experimental data, which indicates that the established computational model is feasible. The contact mechanism between the polishing tool and the workpiece is very complex, and the MRR is affected by the coupling effect of multiple parameters. The above modelling method is a simplified treatment of actual working conditions, and holistic research on the comprehensive effects of the influencing factors is lacking. Furthermore, it is difficult to accurately model the relationship between the MRR and polishing parameters via theoretical modelling methods.

Data-driven models are based on constructing a continuous data loop and feedback mechanism by analysing and mining data related to the prediction target to complete data fusion, autonomous analysis prediction, or diagnosis. Compared with physical models, data-driven models have the advantage of an adaptive ability to correct model predictions. For example, Cai et al. [13] proposed a Gaussian regression process model to dynamically predict the MRR in CMP, and the mean squared error evaluation index of the model reached 3.4. Zhao et al. [14] used a backpropagation (BP) neural network model to predict the effect of magnetic abrasive machining, and the error between their prediction and the actual test result was 7.526%. Neural network technology has the advantages of nonlinear mapping, adaptive learning, parallel processing, fault tolerance, automatic feature extraction, multilevel structure, scalability, and diversity, but there are also problems such as black-box nature, strong dependence on data, overfitting, and poor generalisation ability. With the wide application of machine learning [15], neural network technology plays an important role in process optimisation, especially in the processing of complex, nonlinear process data, can predict the workpiece machining, and, at the same time, can achieve real-time multi-dimensional perception of the machining-process detection.

Therefore, in this study, the influence of processing parameters on the polishing effect is revealed by studying the effects of processing parameters on the magnetorheological–chemical-polishing process. The BP neural network deep learning method is used to predict the polishing results, to construct a prediction model of processing parameters with material removal rate and surface quality, to accurately map the relationship between process parameters and processing quality, and to continuously optimise the process parameters with multi-objective output.

## 2. Experimental Section

### 2.1. Experimental Principle and Device

The principle of magnetorheological–chemical polishing of SiC based on the Fenton reaction is as follows: The Fenton reaction between Fe^2+^ and H_2_O_2_ generates the strong oxidant hydroxyl radical OH, which leads to the oxidative modification of the single-crystal SiC surface. Subsequently, material removal is achieved mechanically via magnetorheological flexible polishing pads. This process is based on the reaction principle (Equations (1)–(3)). During the reaction, Fe^2+^ comes from the weak ionisation of magnetic particles (carbonyl iron powder, CIP) under the action of hydrogen peroxide and does not disappear but only acts as a catalyst. Therefore, the use of carbonyl iron powder as a catalyst does not weaken the magnetorheological effect but rather promotes the Fenton reaction due to the large contact area between the magnetorheological polishing pad and SiC.

(1) $${\mathrm{Fe}}^{2+}+H_{2}O_{2}\to {\mathrm{Fe}}^{3+}+{\mathrm{OH}}^{-}+\cdot \mathrm{OH}$$

(2) $$\mathrm{SiC}+4\cdot \mathrm{OH}+O_{2}\to {\mathrm{SiO}}_{2}+2H_{2}{O+\mathrm{CO}}_{2}$$

(3) $${\mathrm{Fe}}^{3+}+H_{2}O_{2}\to {\mathrm{Fe}}^{2+}+H^{+}+\cdot \mathrm{OOH}$$

**Figure 1:**
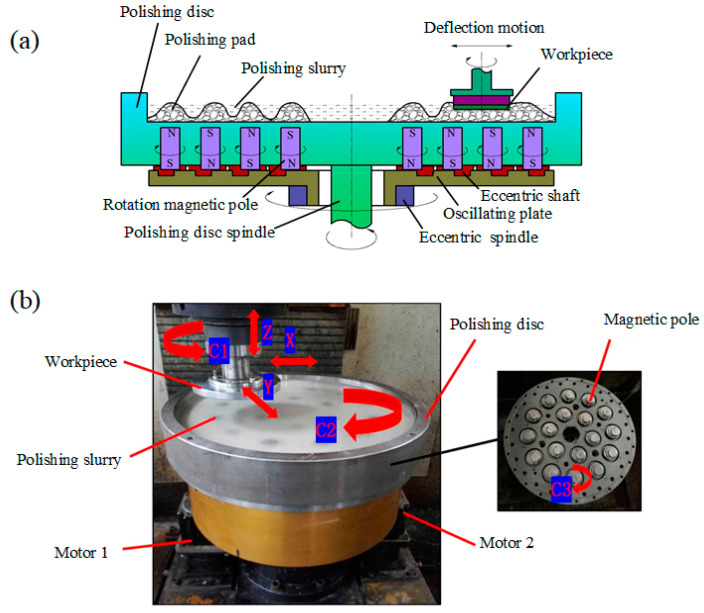
Polishing principle and device. (**a**) processing principle, (**b**) self-constructed device.

The processing principle and self-constructed device used for the test are shown in Figure 1. The entire polishing disc is driven to rotate by its spindle, and a cylindrical small magnet is located under the polishing disc. A magnetorheological polishing slurry consists of carbonyl iron powder, abrasive particles, dispersants, stabilisers, H_2_O_2_, and deionised water, which are used to fill the polishing-disc surface. The magnetic particles in the polishing slurry form a chain string structure under the action of an applied magnetic field, and the abrasive particles are clamped or constrained between the magnetic chain strings to form a flexible magnetorheological effect polishing pad. The workpiece is mounted on the spindle of the machine, and the clearance between the workpiece and the polishing disc is adjusted so that the workpiece contacts the flexible polishing pad and produces relative rotation. During this process, the SiC surface is continuously oxidised and removed, resulting in the efficient processing of SiC.

### 2.2. Experimental Programme

The complex material removal process of magnetorheological–chemical polishing via the Fenton reaction is similar to that of the CMP process, which is affected mainly by the synergistic effect of alternating chemical action and mechanical removal [16,17,18,19]. Only the synergistic effect of these actions can achieve efficient and high-quality processing. To study this effect, the surface roughness Ra and MRR after polishing were evaluated. A diamond was used as an abrasive material (form Zhecheng Hongguang Diamond Technology Co., Ltd., Shangqiu, China), and parameters such as the abrasive grain size, abrasive grain concentration, carbonyl iron powder (CIP) concentration (form Jiangxi Yuean Advanced Materials Co., Ltd., Ganzhou, China), H_2_O_2_ concentration (form Fuzhou Wenlai Biotechnology Co., Ltd., Fuzhou, China), machining gap, workpiece rotational speed, and polishing-disc rotational speed were selected as the research objects to carry out the polishing test. Based on the summary of the previous study and the experience gained from processing other semiconductor materials, the parameter levels were set as shown in Table 1.

Milled 2-inch single-crystal SiC was used as the polished specimen, with a surface roughness Ra of approximately 40 nm (form Beijing TanKeBlue Semiconductor Co., Ltd., Beijing, China). The SiC wafers were weighed before and after polishing via a precision electronic balance with a measurement accuracy of 0.1 mg (OHAUS-CP214, form OHAUS Corporation, Parsippany, NJ, USA), and the MRR was calculated. The morphological characteristics of the processed surface were observed via an OLYMPUS-OLS4000 laser confocal microscope (form Olympus Corporation, Tokyo, Japan). The surface of the polished sample was divided into eight areas, a Mahr XT20 surface roughness profiler (form Mahr GmbH, Gottingen, Germany) was used to measure the Ra of each area of the SiC substrate before and after processing, and the average value of Ra was calculated as an evaluation index of the surface quality before and after polishing.

## 3. Experimental Results

**Figure 2:**
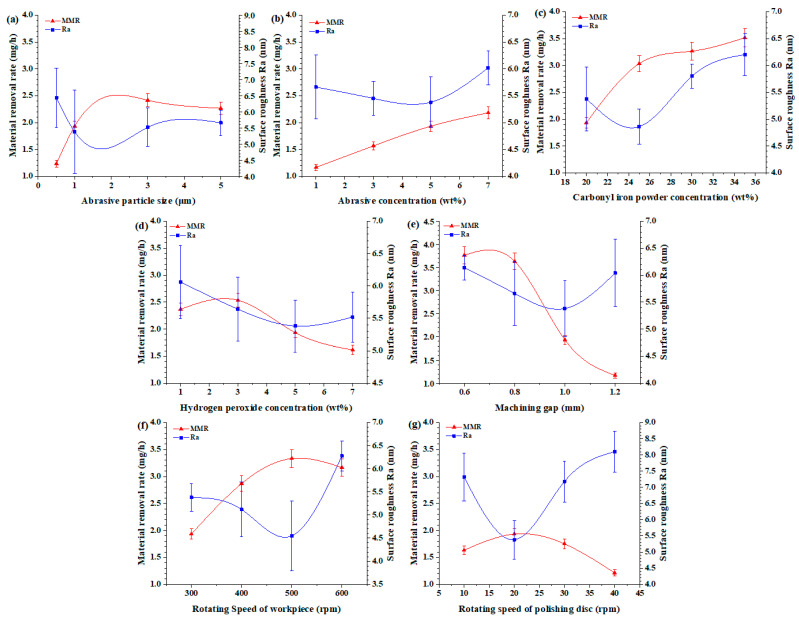
MRR and Ra influence trends of the (**a**) abrasive size, (**b**) abrasive concentration, (**c**) carbonyl iron powder concentration, (**d**) H_2_O_2_ concentration, (**e**) machining gap, (**f**) workpiece speed, and (**g**) polishing-disc speed.

For the polishing of the single-crystal SiC, the experimental results and factor influence patterns are shown in Table 2 and Figure 2.

As shown in Figure 2a, with increasing diamond grain size, the MRR clearly increases, but when the grain size increases to 5 μm, the MRR decreases slightly, whereas the surface roughness Ra tends to decrease and then increase, and the surface roughness decreases when the abrasive grain size is approximately 1 μm. When nanoscale abrasive particles are used, the removal ability of the abrasive material is insufficient because of the large size difference (1.9 μm) with carbonyl iron powder, and the agglomeration of the abrasive material itself seriously affects its processing effect [20]. When the particle size of the abrasive material is comparable to that of carbonyl iron powder, the processing effect is better; however, when the particle size is too large, the magnetorheological polishing pad of the “tolerance effect” is affected [21], the large abrasive particles deteriorate the performance of the magnetorheological polishing pad, the MRR decreases slightly, and the surface roughness increases.

**Figure 3:**
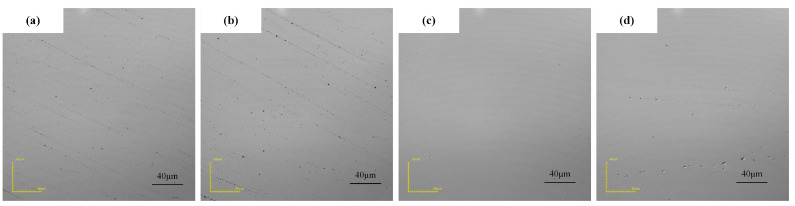
Surface morphology of SiC processed with different abrasive concentrations of (**a**) 1 wt.%, (**b**) 3 wt.%, (**c**) 5 wt.%, and (**d**) 7 wt.%.

As shown in Figure 2b, the MRR increases gradually with increasing abrasive mass fraction, whereas the corresponding change in surface roughness Ra first decreases but then increases. In addition, the surface roughness decreases when the abrasive mass fraction is 5%. Combined with the surface morphology after polishing (Figure 3), when the abrasive concentration is low, the number of effective actions of the abrasive is small, the mechanical removal effect is weak [22], and the grinding scratches remaining in the previous stage of the process are difficult to remove (as shown in Figure 3a,b). With increasing abrasive concentration, the mechanical removal ability increases. When the mechanical removal effect is gradually balanced with chemical action, the best polishing quality is obtained (as shown in Figure 3c). However, when the abrasive concentration exceeds a certain range, the magnetic particles on the abrasive of the constraints are affected [23]. This causes local damage to the surface in the form of pits, resulting in a slight increase in surface roughness (as shown in Figure 3d).

Carbonyl iron powder is one of the most important components in magnetorheological–chemical polishing, on the one hand, as magnetic particles that become a chain string structure under the action of a magnetic field, clamping or restraining the abrasive material to form a magnetorheological polishing pad, and on the other hand, as a catalyst for the chemical reaction to promote the chemistry of the processing process and to soften the SiC surface material. Figure 2c shows the effect of the mass fraction of the carbonyl iron powder on the polishing effect. When the mass fraction of carbonyl iron powder increases, the MRR gradually increases, whereas the surface roughness after polishing tends to decrease but then increases. Moreover, the surface roughness decreases when the mass fraction of carbonyl iron powder is 25%. This result shows that the carbonyl iron powder content determines the material removal ability during magnetorheological–chemical polishing, but too large or too small mass fraction of carbonyl iron powder affects the polishing quality. As mentioned above, the magnetorheological–chemical-compound-polishing process is a synergistic process of chemical action and mechanical removal [7]; within a certain range, the carbonyl iron powder mass fraction increases, the mechanical removal effect increases, and the surface finish is better. However, if the mass fraction of carbonyl iron powder is too large, then the mechanical removal effect produced is much greater than the chemical action, the abrasive scratching effect is enhanced, and the surface roughness is instead increased.

Hydrogen peroxide (H_2_O_2_) is necessary to produce the Fenton reaction, and its concentration determines the kinetics of the Fenton reaction and the amount of hydroxyl radicals generated. Figure 2d shows that as the mass fraction of hydrogen peroxide increases, the MRR first increases and then decreases, whereas the surface roughness Ra shows the opposite trend. The H_2_O_2_ concentration affects the rate of ·OH generation (as shown in Equation (1)), which affects the rate of generation of the SiO_2_ oxide layer on the surface of SiC, which in turn affects the polishing effect [24]. When the H_2_O_2_ mass fraction is small, the generation of hydroxyl radicals fails to fully oxidise with the SiC surface material, affecting the polishing effect. As the hydrogen peroxide mass fraction increases, the generation of hydroxyl radicals increases, the thickness of the oxide layer on the surface of the SiC increases, the MRR increases, and the surface roughness decreases. When the hydrogen peroxide mass fraction reaches a certain value, the hydrogen peroxide captures the generated hydroxyl radicals, hindering the effective oxidation of SiC [25]; the reaction formula is expressed via Equation (4). Moreover, excessive hydrogen peroxide will cause a large amount of Fe^2+^ to be oxidised to Fe^3+^ and form flocculent precipitates [Fe(H_2_O)_n_(OH)_6-n_]_3-n_ [26], as described via Equation (5). These precipitates affect the chemical reaction effect, resulting in reduced material removal rates and increased surface roughness.

(4) $$H_{2}O_{2}+\cdot \mathrm{OH}\to H_{2}O+\cdot \mathrm{OOH}$$

(5) $${\mathrm{Fe}}^{3+}+3{\mathrm{OH}}^{-}\to \mathrm{Fe}{\left(\mathrm{OH}\right)}_{3}↓$$

**Figure 4:**
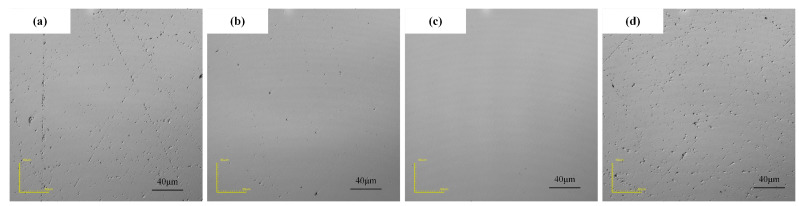
Surface morphology of SiC with different machining gaps of (**a**) 0.6 mm, (**b**) 0.8 mm, (**c**) 1.0 mm, and (**d**) 1.2 mm.

The machining gap determines the magnetic field strength in the machining area and the contact state between the magnetorheological polishing pad and the workpiece surface. Figure 2e shows that when the machining gap increases, the MRR decreases continuously, while the corresponding surface roughness decreases and then increases. Additionally, a lower surface roughness is obtained at a machining gap of 1.0 mm. This shows that it is necessary to appropriately select the machining gap for magnetorheological polishing. Changing the machining gap directly affects the magnetic field strength in the machining area, which in turn affects the polishing pressure on the surface of the workpiece [27]. As the processing gap increases, the magnetic induction strength gradually decreases, and the effect of the magnetorheological polishing pad on the workpiece decreases, i.e., the smaller the machining gap, the greater the material removal rate, and the lower the surface roughness. However, when the machining gap is too small, because the polishing force effect is greater, but surface scratches appear, the machining surface roughness increases. As shown in Figure 4, for the surface morphology of the machined workpiece, when the machining gap is 1.0 mm, the machined surface roughness is lower (Figure 4c).

Figure 2f,g show the results for different workpiece speeds and polishing-disc speeds, respectively. That is, the MRR and surface roughness of the two groups show the same trend. As the workpiece speed and the polishing-disc speed increase, the material removal rates of the two groups first increase but then decrease, whereas the surface roughness tends to first decrease but subsequently increases. Clearly, there is an optimum relative speed parameter in the machining process. The rotational speed of the workpiece and the polishing disc affects the number of times the abrasive acts on the workpiece per unit of time, which is an important parameter for material removal. According to the Preston equation [28], the MRR is directly proportional to the rotational speed, and an increase in the relative speed between the workpiece and the abrasive grain within a certain range leads to an increase in the mechanical action of the abrasive, which in turn increases the MRR. However, as the workpiece speed and polishing-disc speed continue to increase to a certain extent, high-speed relative motion decreases the stability of the magnetorheological polishing pad, which, to a certain extent, weakens the processing effect [29]. Moreover, too high of a relative speed will lead to the centrifugal separation of the workpiece slurry on the polishing disc. In particular, the escape phenomenon that occurs when the density of abrasive particles is different from that of magnetorheological particles causes a reduction in the number of effective abrasive particles involved in polishing and a decrease in the material removal rate. Related research also shows that the magnetorheological fluid has an obvious shear thinning effect [30,31]; that is, as the relative rotational speed increases, the viscosity of the magnetorheological fluid decreases, thus reducing the polishing pressure of the polishing pad on the workpiece. Therefore, magnetorheological polishing should not be carried out at too high a relative speed.

## 4. Prediction Model Based on the BP Neural Network

### 4.1. BP Neural Network Modelling

The experimental results indicate that the polishing process is affected by multiple parameters, such as the polishing slurry and processing technology, and that there are interactions between the parameters, thus affecting the synergistic process of chemical and mechanical effects. Thus, it is difficult to directly reflect or predict the mapping relationship between each parameter variable and the polishing results through theoretical formulas. The BP neural network can be trained and learn on its own, combined with the given input value to obtain the closest results of the desired output value. Therefore, the BP neural network method is used to systematically train and learn from the experimental results to establish a prediction model for SiC magnetorheological polishing material removal and surface roughness.

The BP neural network mainly contains an input layer, a hidden layer, and an output layer. The parameters of the abrasive grain size, abrasive grain concentration, carbonyl iron powder concentration, H_2_O_2_ concentration, machining gap, workpiece rotational speed, and polishing-disc rotational speed are taken as the input layers of the neural network model; i.e., the number of nodes in the input layer is seven. The MRR and the roughness of the polished surface are taken as the output layer; i.e., the number of nodes in the output layer is two. The number of hidden layers influences the complexity and expressive ability of the neural network, and there is no clear selection criterion for the number of nodes in the hidden layer; however, it is related to the number of nodes in the input/output layer. Different empirical formulas are usually used to guide the selection of the number of nodes of the hidden layer, and in the actual selection, an approximate range is determined via Equations (6)–(8), and the optimal number of nodes is subsequently determined via a trial-and-error method [32,33,34].

(6) l≥2n

(7) $$l=\sqrt{m+n}+a$$

(8) l=2n+1

where *l* is the number of nodes in the hidden layer, *n* is the number of nodes in the input layer, *m* is the number of nodes in the output layer, and *a* is a constant between 0 and 10.

To increase the training speed and improve the model performance, stability, and generalizability, the data samples are normalised so that the sample data containing different magnitudes are transformed into dimensionless data. In addition, the data are restricted to the [0, 1] interval, as expressed via Equation (9) [35]. The normalised data are processed from the input layer to the output layer; therefore, the output needs to be anti-normalised, as expressed via Equation (10).

(9) $$x_{k}=\frac{x-x_{\min}}{x_{\max}-x_{\min}}$$

(10) $$y=y_{k}\times (x_{\max}-x_{\min})+x_{\min}$$

where *x_k_* is the normalised value, *x* is the sample data, *x_min_* is the minimum value in the sample data, *x_max_* is the maximum value in the sample data, *y* is the inverted normalised value, and *y_k_* is the normalised value of the predicted output.

**Figure 5:**
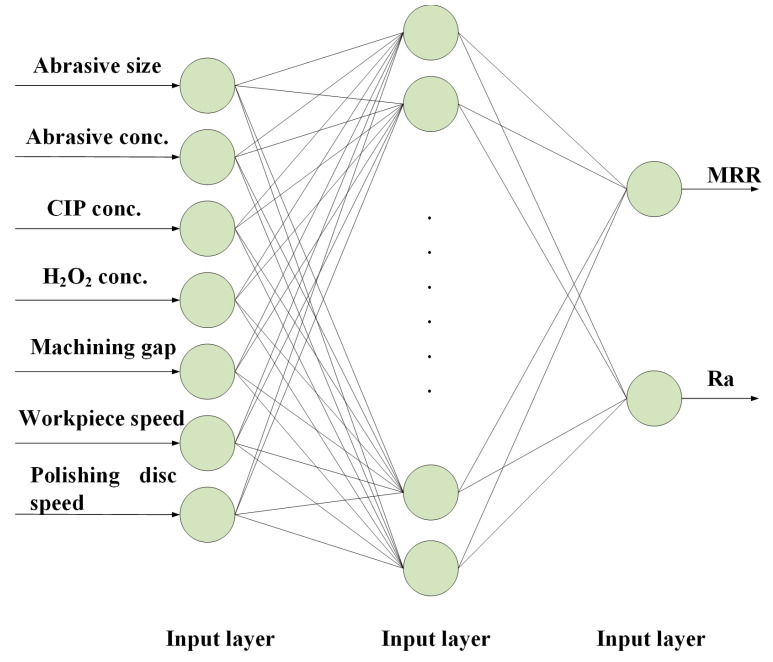
BP neural network structure.

MATLAB 9.8 is used to construct the BP neural network model. Selecting the number of hidden nodes in the range of 13–16 and comprehensively comparing the performance of the number of nodes in each hidden layer, it is found that the fastest prediction effect can be achieved when the number of hidden nodes is 15, so the number of nodes in the hidden layer is selected to be 15; i.e., the structure of the neural network is established to be 7-15-2, as shown in Figure 5. The experimental results of groups No. 1–16 are used as training samples, and the experimental results of groups No. 17–22 are used as test samples. The maximum number of iterations is set to 10,000, the training accuracy is 0.0001, the learning rate is 0.01, and the maximum number of failures is 200. The tanh function is adopted as the activation function of the hidden layer, as expressed via Equation (11), and the sigmoid function is used as the output layer activation function, as expressed via Equation (12). The Adam optimisation algorithm is used as a backpropagation function [36].

(11) $$\tanh(x)=\frac{\sinh(x)}{\cosh(x)}=\frac{e^{x}-e^{-x}}{e^{x}+e^{-x}}$$

(12) $$\mathrm{sigmod}(x)=\frac{1}{1+e^{-x}}$$

### 4.2. Forecast Results and Analyses

The MRR and surface roughness of the polishing process were predicted via a BP neural network, and the results of the comparison between the measured values (MV) and the predicted values (PV) and the relative error values (RE) are shown in Table 3. As shown in this table, the predicted output values are very close to the measured values, and the training error values of the MRR and surface roughness are within 9% and 3%, respectively, whereas the corresponding test validation error values are within 4%, which may be due mainly to the insufficient number of samples, and the prediction model’s error can be further reduced if the number of samples is increased.

To further analyse the prediction ability of this prediction model, the mean absolute percentage error (MAPE), the coefficient of determination R^2^, and root mean squared error (RMSE), are introduced, which are expressed via Equations (13), (14) and (15), respectively, and the results are shown in Table 4. The total MAPE of the prediction model for the MRR and surface roughness is less than 2% and 1%, respectively, the coefficient of determination R^2^ is close to 1, and the RMSE values are relatively small. This finding shows that the prediction model has high accuracy and precision and can effectively predict the effect of magnetorheological polishing of SiC via the Fenton reaction, which provides a new idea for the optimisation of the polishing process.

(13) $$\delta_{MAPE}=\frac{100\%}{n}\sum_{i=1}^{n}\left|\frac{y_{i}-y_{i}^{*}}{y_{i}^{*}}\right|$$

(14) $$R^{2}=1-\frac{\sum_{i=1}^{n}{\left(y_{i}-y_{i}^{*}\right)}^{2}}{\sum_{i=1}^{n}{\left(y_{i}^{*}-{\bar{y}}^{*}\right)}^{2}}$$

(15) $$RMSE=\sqrt{\frac{\sum_{i=1}^{n}{\left(y_{i}-y_{i}^{*}\right)}^{2}}{n}}$$

where *y_i_* is the output prediction, $y_{i}^{*}$ is the true value of the measurement, y^*^ is the mean value of $y_{i}^{*}$, and *n* is the total number of samples.

## 5. Conclusions

(1) Magnetorheological–chemical composite polishing is a process of mechanical removal coupled with chemical action, and the polishing effect is better only when the two actions are synergistic.
(2) A large abrasive particle size affects the “tolerance effect” of the polishing pad, and better results are obtained when the abrasive particle size is close to the size of the carbonyl iron powder. The abrasive mass fraction affects the ability of the magnetic particles to constrain the abrasive particles, thus affecting their processability. A suitable oxidant mass fraction exists for the Fenton reaction process, and too large a mass fraction of hydrogen peroxide will instead result in a burst reaction. The machining gap affects the magnetic field strength in the machining area, which in turn affects the polishing pressure on the surface of the workpiece. The workpiece and polishing disc rotational speed affect the stability of the polishing pad, and too high a relative speed results in the heart separation phenomenon.
(3) When the BP neural network is used to construct a prediction model of the MRR and surface quality, its average absolute percentage error is less than 2%, which is important for the online monitoring and process optimisation of the machining process.

## Figures and Tables

**Table 1:** Machining parameters and their levels.

Research Objects	Parameter Levels
Abrasive size (μm)	0.5, 1.0, 3.0, 5.0
Abrasive concentration (wt%)	1, 3, 5, 7
CIP concentration (wt%)	20, 25, 30, 35
H_2_O_2_ concentration (wt%)	1, 3, 5, 7
Machining gap (mm)	0.6, 0.8, 1.0, 1.2
Workpiece speed (rpm)	300, 400, 500, 600
Polishing disc speed (rpm)	10, 20, 30, 40

**Table 2:** SiC polishing experimental conditions and results.

No.	Abrasive Size (μm)	Abrasive Conc. (wt%)	CIP Conc. (wt%)	H_2_O_2_ Conc. (wt%)	Machining Gap (mm)	Workpiece Speed (rpm)	Polishing Disc Speed (rpm)	MRR (mg/h)	Ra (nm)
1	0.5	5	20	5	1	300	20	1.23	6.44
2	1	5	20	5	1	300	20	1.93	5.38
3	3	5	20	5	1	300	20	2.42	5.52
4	5	5	20	5	1	300	20	2.27	5.66
5	1	1	20	5	1	300	20	1.17	5.66
6	1	3	20	5	1	300	20	1.57	5.45
7	1	7	20	5	1	300	20	2.18	6.02
8	1	5	25	5	1	300	20	3.03	4.86
9	1	5	30	5	1	300	20	3.27	5.80
10	1	5	35	5	1	300	20	3.52	6.20
11	1	5	20	1	1	300	20	2.37	6.06
12	1	5	20	3	1	300	20	2.53	5.64
13	1	5	20	7	1	300	20	1.62	5.52
14	1	5	20	5	0.6	300	20	3.77	6.14
15	1	5	20	5	0.8	300	20	3.63	5.66
16	1	5	20	5	1.2	300	20	1.17	6.04
17	1	5	20	5	1	400	20	2.87	5.12
18	1	5	20	5	1	500	20	3.33	4.55
19	1	5	20	5	1	600	20	3.17	6.28
20	1	5	20	5	1	300	10	1.63	7.32
21	1	5	20	5	1	300	30	1.75	7.18
22	1	5	20	5	1	300	40	1.21	8.10

**Table 3:** Comparison of the measured values with neural network predictions.

Training Samples							Test Samples						
	MRR (mg/h)			Ra (nm)				MRR (mg/h)			Ra (nm)		
No.	ME	PE	RE (%)	ME	PE	RE (%)	No.	ME	PE	RE (%)	ME	PE	RE (%)
1	1.23	1.2326	−0.06	6.44	6.4345	−0.08	17	2.87	2.9402	2.53	5.12	4.9304	−3.70
2	1.93	1.8009	−6.85	5.38	5.5182	2.57	18	3.33	3.4571	3.71	4.55	4.6631	2.49
3	2.42	2.4157	−0.02	5.52	5.5188	−0.02	19	3.17	3.1671	0.01	6.28	6.2789	−0.02
4	2.27	2.2668	0.00	5.66	5.6589	−0.02	20	1.63	1.6127	−1.26	7.32	7.2279	−1.26
5	1.17	1.1687	0.17	5.66	5.6599	0.00	21	1.75	1.7517	0.01	7.18	7.1749	−0.07
6	1.57	1.4304	−8.70	5.45	5.4881	0.70	22	1.21	1.2124	−0.06	8.1	8.0969	−0.04
7	2.18	2.1786	−0.22	6.02	6.0200	0.00							
8	3.03	3.0340	0.02	4.86	4.8563	−0.08							
9	3.27	3.2106	−1.72	5.8	5.6894	−1.91							
10	3.52	3.5151	−0.03	6.2	6.1974	−0.04							
11	2.37	2.3659	−0.03	6.06	6.0589	−0.02							
12	2.53	2.5338	0.02	5.64	5.6379	−0.04							
13	1.62	1.6169	0.04	5.52	5.5139	−0.11							
14	3.77	3.7652	−0.04	6.14	6.1396	−0.01							
15	3.63	3.6291	−0.06	5.66	5.6596	−0.01							
16	1.17	1.1669	0.02	6.04	6.0344	−0.09							

**Table 4:** Errors in the results of the prediction model.

		δMAPE/(%)	R^2^	RMSE
MRR	Training	1.13%	0.997	0.050
	Test	1.52%	0.995	0.065
	Total	1.16%	0.996	0.052
Ra	Training	0.36%	0.985	0.045
	Test	1.51%	0.994	0.107
	Total	0.60%	0.993	0.064

## Data Availability

The original contributions presented in this study are included in the article. Further inquiries can be directed to the corresponding author.
